# Diseases of Overfed Convicts

**Published:** 1856-07

**Authors:** 


### THE DISEASES OF OVERFED CONVICTS.*

It is a most remarkable fact, and one that will long have record in English history, that at or about the time when our Crimean army of martyrs was suffering all the horrors of a *famine plague,* another body of Englishmen, not martyrs, but outcasts, on t'other side earth, were undergoing the miseries of what may be called a *gluttony plague.* Thus do extremes meet,—and so is there but one move from the sublime to the ridiculous.

Let us break the enigma. At the close of the year 1854, a period ever memorable in connexion with the starvation of our army, Dr. Rennie, a medical officer in the Queen's service, having charge of the convict establishment at Fremantle, in Western Australia, was busily occupied in drawing up an earnest and convincing report to his superior officer. His object was to prove that the bodies of the convicts placed under his care were—oh, inhuman thought! the victims of a system of Epicurean philosophy, so rigidly carried out, that for month after month the unhappy men, catching, as it were, an infection from the tables at which they—shall we say it? —crammed, groaned heavily under the weight of a perennial feast, such as few human lips have laboured at or human stomachs surfeited from.

On each side earth, in fact, at the same time, a grand physiological experiment was being unconsciously conducted. In the cold winter of the Crimea a band of heroes were suffering the horrors of starvation, coupled with overwork. In the warm and genial climate of Australia, a band of vagabonds were undergoing the miseries arising from a systematic process of *overfeeding,* coupled with deficiency of exertion. Destitution and luxury were each doing their worst work on the largest scale.

Content with recording this remarkable coincidence, we proceed to the narration of the *glattony plague* as described in Dr. Rennie's interesting and thoughtful report.

From the 1st of July, 1854, to 31st of December of the same year, there came under medical treatment at the convict establishment at Fremantle, 1,554 patients. Of these, the vast majority suffered from one or other of three classes of disease, viz., diseases of the digestive organs, inflammatory affections of the eyes, and cutaneous eruptions; they

* Further Correspondence on Convict Discipline and Transportation. Blue Book. London: 1856.

were all, in the opinion of Dr. Rennie, the results of over-feeding, assisted occasionally by a deficiency of vegetable acids. The amount of food daily supplied to each probation convict, was at the end of 1854 as follows. *Breakfast*, 12 oz. of bread, 1 pint of tea, with $\frac{3}{4}$ oz. of sugar and $\frac{1}{6}$ oz. of tea ; total weight, 2 lbs. *Dinner*, 16 oz. of potatoes, 16 oz. of meat, 6 oz. of bread, $\frac{1}{2}$ oz. of salt, $\frac{1}{4}$ oz. of pepper, 1 pint of soup, and 1 oz. of rice, barley, or oatmeal for thickening the soup ; total, 3 lbs. 10 oz. *Supper*, 8 oz. of bread, 1 pint of tea, as for breakfast ; total, 1 lb. 12 oz. The total weight of food allowed per diem was thus seven pounds six ounces, including fifty-nine ounces of solid aliment; while to the blacksmiths and sawyers, an extra ration of 4 oz. of bread and 4 oz. of meat was given, bringing their diet up to 7 lbs. 14 oz., of which 67 oz. was solid aliment.

In support of his view, that upon the excess of diet the peculiar diseases to which the prisoners were subject were mainly dependent, Dr. Rennie offered the following reasonable proofs.

1. That prisoners who had been some time *re*-convicted, and, consequently, habituated to a lower scale of diet than the probation prisoners, enjoyed much the best health.

2. That probation prisoners, when temporarily placed on low diet, rapidly improved in general health.

3. That medicines, without a considerable reduction in diet, exercised little or no permanent influence over the ailments of the prisoners.

4. That after every species of treatment had failed in arresting dysentery, cutaneous diseases, and ophthalmia, especially the latter, a moderate and properly regulated diet, with a sufficiency of vegetable acid, speedily effected a cure.

5. That the want of vegetable acid in the diet, was proved by the temporary improvement which resulted from the free use of lime-juice, even when no reduction of diet took place, as in the out-door treatment of some cutaneous eruptions.

6. That, irrespective of the evidence furnished by the amount of disease, a comparison of the diet supplied to the convicts with that which is considered necessary in England for a man exposed to the ordinary vicissitudes of a labourer's life, was sufficient to prove how much the diet was in excess of what is either requisite or safe ; the diet furnished to the probation prisoners exceeding by one pound, *after allowing for loss by cooking*, that which is calculated by competent authorities for a labouring man in England ; and this excess being also supplied in a climate where the chemical demands of the system for food are much less, and where an over supply must, from physical laws, end in the production of functional, and its natural sequence, organic disease.

7. That the extraordinary prevalence of constipation, a prevalence altogether inconsistent with health, was a strong proof that the diet exceeded the systemic demands for food.

8. That the diseases so prevalent amongst the prisoners were almost entirely unknown amongst the troops, the pensioners, many of whom were debilitated from long service, and the population generally in Fremantle, although these classes were exposed to the same exciting causes as the prisoners were, save and except in the matter of diet.

9. That in the medical treatment of the convicts nothing succeeded so well as the administration of purgative medicines in doses enormously large (say five drops of croton oil or sixty grains of jalap); that, under such medical influence, the quantity of alvine matter thrown off was sometimes incredible, amounting, in one case, to not less than thirty pounds; and that this excrete was always more or less in a state of decomposition or fermentation.

Such were some of the positive proofs in support of his theory brought forward by Dr. Rennie; but he went a step further. He showed that there was an absence of other causes. He pointed out that the atmosphere was pure; that the general locality of Fremantle was favourable to health, and that the diseases prevailing amongst the prisoners were altogether unknown amongst the community generally; that the site of the establishment was good; that, as the convicts during their daily labour were exposed to the same external agencies as the sappers and miners, warders, and free mechanics, all of whom escaped from the convicts' diseases, a miasmatic influence could not be entertained as an exciting cause; that the wards of the convict establishment were well ventilated, being in this respect much superior to the wards of the barracks where the soldiers were lodged; that there was no evidence of the diseases named having spread by contagion, the orderlies of the hospital all having good health: that the water supply was excellent; that, in fact, reduction to punishment diet of bread and water for some days effected a cure; that moral depression, from the position of the men as prisoners, was removed out of the field of argument, by the circumstance that 80 per cent. of them were better off than they ever were in their lives before, while those most exposed to such depressing influences, viz., those under solitary confinement with reduced diet, enjoyed perfect immunity from the diseases in question, and had far better health than the rest of the prisoners.

Having thus, both by affirmative and negative evidence, proved his position, Dr. Rennie proceeded to point out that the type of the diseases to which he specially referred, was of a scrofulous or gouty character, resulting from disordered digestion and from an amount of overwork thrown on the natural excreting organs; and ultimately he begged, for the physical security of his convict excellencies, that their provender may be reduced to the following modest scale:—

| *Breakfast.* | *Dinner.* | *Supper.* |
|---|---|---|
| 10 oz. of bread. | 12 oz. of meat. | 8 oz. of bread. |
| 1 pint of tea. | 1 lb. of potatoes. | 1 pint of tea. |

That is to say, that their solid food might be reduced to forty-six ounces a day; a scale ten ounces above what is required in England for the complete nourishment of a labouring man working twelve hours a day, and in a climate requiring a greater proportion of food.

In the conclusion of his Report, Dr. Rennie drew attention to the fact, that an abundance of excellent fish exists in the waters about Fremantle, and suggested that the prisoners should be employed in catching them. "Thus", says he innocently enough, "the prisoners would to a certain extent diminish the cost of their support;" and a large gang of men might find employment, "who, from long continued indigestion, have become quite unfit for any labour entailing much physical exertion, and to find employment for whom is a constant source of difficulty to the superintendent."

The same class of men abounds also at the depôts, where, if no scope is allowed them for piscatorial pursuits, they might be employed in manufacturing arrowroot from the zamia plant. "The process is simple, and the farina procured equal to the best kinds of Indian arrowroot. At Freshwater Bay depôt, the men *amuse themselves* in the evening making it, fourteen pounds of the central portion of the root making a pound and a quarter of arrowroot in three or four hours." Indeed, unbounded resources exist; and the encouragement of the manufacture of arrowroot at the depôts might develope amongst the prisoners a mode of earning their livelihood, and lead to the establishment of a new and important article of commerce and export.

According to Dr. Rennie, the saving to the crown which would arise from reducing the diet roll to the scale he suggested, would amount to at least £10,000 per annum.

But it was in a physiological point of view that the question required to be most carefully reviewed. With great care and ability our author analysed the well considered arguments of Dr. Andrew Combe and Sir James Clark on the subject of digestion. He showed that the evils of overeating are virtually the same and nearly as serious as those of privation; that there is a limit to the powers of the natural secreting and excreting organs; that under an excess of overwork these organs cease to act in great part; and that upon this, various derangements, amongst which dyspepsia is the prominent one, inevitably stand forth.

Dr. Combe, in his *Physiology of Digestion*, remarks specially on a symptom of gnawing sensation in the stomach and craving for more food, which is so common an indication of irritable stomach and indigestion arising from overfeeding.

With cases of this character, the Fremantle convict establishment seems to have been overflowing prior to the appearance of the report now in hand; and Dr. Rennie records many striking examples of this kind. We will sketch out one only. On the 16th of February, 1854, a convict named John Hudson, register number 3,035, was admitted into hospital labouring under an aggravated form of indigestion, characterised by great uneasiness in the stomach, cough, and difficulty in swallowing. On examination, the throat and gullet were found in a state of chronic inflammation, and throwing off a profuse purulent discharge. On visiting the infirmary at noon on the day of his admission, Dr. Rennie found Mr. Hudson comfortably ensconced behind the following trifling repast,—nine ounces of solid meat, eight ounces of bread, twenty-one ounces of potatoes and cabbage, with one pint of soup thickened with oatmeal, and weighing twenty ounces; thus completing arrangements for the taxation of a grossly disordered stomach, to the extent of fifty-eight ounces of food, at one inevitable go. If to this collation breakfast and supper sustenance be added, it follows that Mr. Hudson was consuming daily seven pounds of food, a proportion, if his absolute weight amounted to twelve stones or one hundred and sixty-eight pounds, equivalent to a twenty-fourth part of his whole body. Granting also for something considerable in the way of loss, it would follow further, that if this gentleman's assimilating and wasting powers had been commensurate with his reception faculties, he would virtually have exchanged the elements of his body and have assumed an entire new corporeal development every lunar month at least; a calculation upon which an original mode of reforming criminals might possibly be based, could the mental constitution be made, by some apposite discovery, to change *pari passu* with the physical. This latter point commands respectful consideration. Unfortunately, however, in the present case, even the physical waste of our victim would not run on in proportion to his supplies; so he came under medical care, and, in spite of his strongest remonstrances, which showed the decision of his character, he was put on low diet and soon recovered.

And now the reader will naturally ask, Why are convicts thus over replenished? The answer is, that the authorities find that a body of men are more easily managed when well clad, well lodged, and supplied with more food than will satisfy their animal cravings. Treated on this principle, they can readily be punished by the withdrawal of their luxuries. This is the only argument. Whether it is a justifiable mode of attaining the object in view, opens, says Governor Kennedy, "a most serious question."—Serious, indeed, after Dr. Rennie's clear and plain-spoken revelations.

The report of Dr. Rennie met with some opposition from his superior, Dr. Galbraith, who evidently has no leanings to common sense physiological deductions. However, in the establishment at Fremantle, the main point was ultimately carried. The diet allowance was lowered; and a second report, bearing date June 30, 1855, states that the reduced diet, though still capable of further reduction, answered well, and that the hospital records afforded convincing proofs that a very great change for the better in the general health of the ticket of leave men and the prisoners had taken place, since the reduced ration had been in operation.

In a sanitary as well as in a political point of view, the value of such facts as are above recorded cannot be over estimated. In the world at large we see too frequently what the effects of starvation are on a comprehensive scale; but it is a novelty, both in physiology and pathology, to get a wholesale view of the effects of over repletion from one continued form of diet. A modern Magendie, wishing to investigate further into the subject of the food elements that build up, and the food elements that keep the animal fire alive, need sacrifice no more unhappy dogs at the shrine of Æsculapius. Let him but read in English blue books how, with an eye to the progress of physiological science, our authorities overfeed, underfeed, and don't feed at all, their convicts, their fighting men, and their paupers; and he will find sufficient facts ready made to afford him full occupation in arrangement and deduction for five years at least.

